# Supplementary material for: Diagnostic Efficacy of Serological Antibody Detection Tests for Hepatitis Delta Virus: A Systematic Review and Meta-Analysis
Source: Viruses. 2023 Nov 29;15(12):2345. doi: 10.3390/v15122345 (PMC10747714; doi:10.3390/v15122345)
Supplement: Supplementary file 1 [file viruses-15-02345-s001.zip › Supplementary figures.pdf]

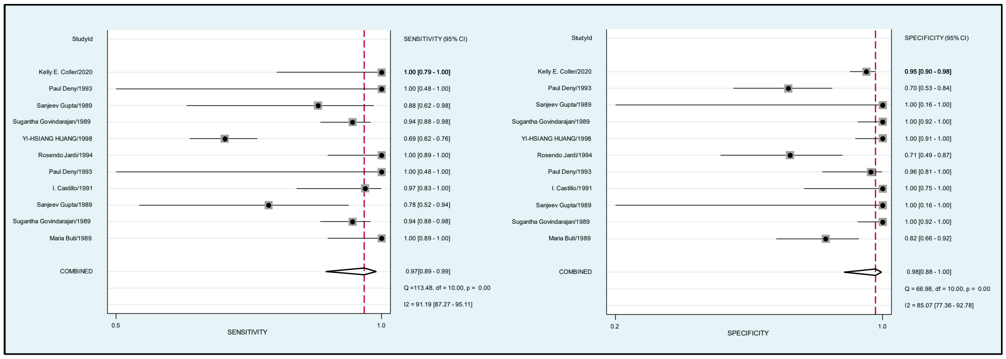

Figure S1 Forest plots of the pooled sensitivity and specificity for anti-HDV IgG or IgM antibodies in diagnosis of HDV.

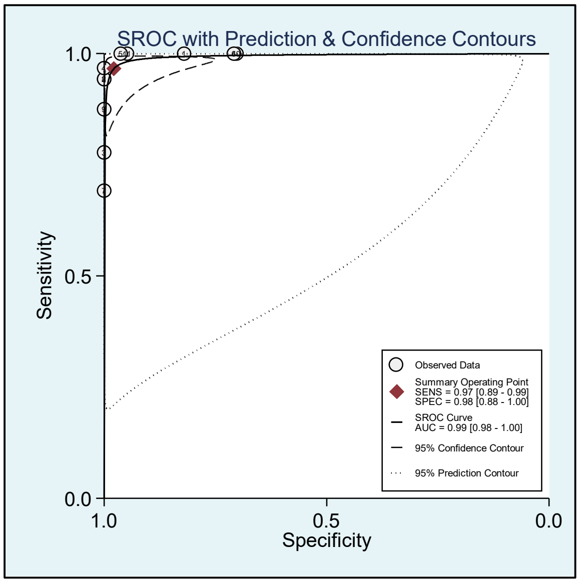

Figure S2 The SROC curves of the serological testing of anti-HDV IgG or IgM antibodies**.**
