# Supplementary material for: Diagnostic Efficacy of Serological Antibody Detection Tests for Hepatitis Delta Virus: A Systematic Review and Meta-Analysis
Source: Viruses. 2023 Nov 29;15(12):2345. doi: 10.3390/v15122345 (PMC10747714; doi:10.3390/v15122345)
Supplement: Supplementary file 1 [file viruses-15-02345-s001.zip › Supplementary File S1.pdf]

Supplementary File S1. Search Strategy

Search Database: PubMed

Publication date: January 1, 1989, to May 31, 2023.

Search Strategy: ((Hepatitis D[MeSH Terms]) OR (hepatitis delta virus[MeSH Terms]))AND ((serologic tests[MeSH Terms]) OR (Diagnosis[MeSH Terms]) OR (Enzyme-Linked Immunosorbent Assay[MeSH Terms]) OR (Immunoglobulin G[MeSH Terms]) OR (Immunoglobulin M[MeSH Terms])) AND ((Sensitivity and Specificity[MeSH Terms]) OR (Data Accuracy[MeSH Terms]) OR (efficacy)) AND ("1989/01/01"[Date - Publication] : "2023/05/31"[Date - Publication])

Search Database: Cochrane

Publication date: January 1, 1989, to May 31, 2023.

Search Strategy: #1 MeSH descriptor: [Hepatitis D] explode all trees 89

#2 (Infection, Delta):ti,ab,kw OR (Hepatitis, Delta):ti,ab,kw OR (Delta Hepatitis):ti,ab,kw OR (Hepatitides, Delta):ti,ab,kw OR (Delta Infection):ti,ab,kw OR (Infections, Delta):ti,ab,kw OR (Superinfection, Delta):ti,ab,kw OR (Delta Superinfections):ti,ab,kw OR (Superinfections, Delta):ti,ab,kw OR (Delta Superinfection):ti,ab,kw OR (Amazon Black Fever):ti,ab,kw OR (Fever, Amazon Black):ti,ab,kw OR (Labrea Disease):ti,ab,kw OR (Disease, Labrea):ti,ab,kw OR (Diseases, Labrea):ti,ab,kw OR (Labrea Diseases):ti,ab,kw OR (Black Fever, Amazon):ti,ab,kw with Cochrane Library publication date Between Jan 1989 and May 2023, in Trials 647

#3 MeSH descriptor: [Hepatitis Delta Virus] explode all trees 28

#4 (Delta Virus, Hepatitis):ti,ab,kw OR (Delta Viruses, Hepatitis):ti,ab,kw OR (Hepatitis Delta Viruses):ti,ab,kw OR (Hepatitis D Virus):ti,ab,kw OR (Hepatitis D Viruses):ti,ab,kw OR (Delta Virus):ti,ab,kw OR (Delta Viruses):ti,ab,kw OR (Delta Agent):ti,ab,kw OR (Delta Agents):ti,ab,kw OR (Deltavirus):ti,ab,kw OR (Deltaviruses):ti,ab,kw with Cochrane Library publication date Between Jan 1989 and May 2023 2265

#5 MeSH descriptor: [Serologic Tests] explode all trees1807

#6 (Serologic Tests):ti,ab,kw OR (Tests, Serologic):ti,ab,kw OR (Test, Serological):ti,ab,kw OR (Test, Serologic):ti,ab,kw OR (Serological Tests):ti,ab,kw OR (Serological Test):ti,ab,kw OR (Serologic Test):ti,ab,kw OR (Tests, Serological):ti,ab,kw OR (Serodiagnosis):ti,ab,kw OR (Serodiagnoses):ti,ab,kw with Cochrane Library publication date Between Jan 1989 and May 2023 1721

#7 MeSH descriptor: [Diagnosis] explode all trees 446946

#8 (Diagnoses):ti,ab,kw OR (Diagnose):ti,ab,kw OR (Diagnoses and Examinations):ti,ab,kw OR (Examinations and Diagnoses):ti,ab,kw OR (Diagnoses and Examination):ti,ab,kw OR (Examination and Diagnoses):ti,ab,kw OR (Postmortem Diagnosis):ti,ab,kw OR (Diagnoses, Postmortem):ti,ab,kw OR (Diagnosis, Postmortem):ti,ab,kw OR (Postmortem Diagnoses):ti,ab,kw OR (Antemortem Diagnosis):ti,ab,kw OR (Antemortem Diagnoses):ti,ab,kw OR (Diagnoses, Antemortem):ti,ab,kw OR (Diagnosis, Antemortem):ti,ab,kw with Cochrane Library publication date Between Jan 1989 and May 2023 187236

#9 MeSH descriptor: [Enzyme-Linked Immunosorbent Assay] explode all trees 2699
#10 (Immunosorbent Assays, Enzyme-Linked):ti,ab,kw OR (Enzyme Linked Immunosorbent Assay):ti,ab,kw OR (Assay, Enzyme-Linked Immunosorbent):ti,ab,kw OR (ELISA):ti,ab,kw OR (Assays, Enzyme-Linked Immunosorbent):ti,ab,kw OR (Enzyme-Linked Immunosorbent Assays):ti,ab,kw OR (Immunosorbent Assay, Enzyme-Linked):ti,ab,kw with Cochrane Library publication date Between Jan 1989 and May 2023 16711
#11 MeSH descriptor: [Immunoglobulin G] explode all trees 4573
#12 (IgG2B):ti,ab,kw OR (Allerglobuline):ti,ab,kw OR (IgG1):ti,ab,kw OR (IgG(T)):ti,ab,kw OR (GT, Immunoglobulin):ti,ab,kw OR (Immunoglobulin GT):ti,ab,kw OR (Polyglobin):ti,ab,kw OR (IgG2):ti,ab,kw OR (IgG2A):ti,ab,kw OR (Gamma Globulin, 7S):ti,ab,kw OR (IgG Antibody):ti,ab,kw OR (Antibody, IgG):ti,ab,kw OR (IgG):ti,ab,kw OR (7S Gamma Globulin):ti,ab,kw OR (IgG4):ti,ab,kw OR (IgG3):ti,ab,kw with Cochrane Library publication date Between Jan 1989 and May 2023 8730
#13 MeSH descriptor: [Immunoglobulin M] explode all trees637
#14 (19S Gamma Globulin):ti,ab,kw OR (Gamma Globulin, 19S):ti,ab,kw OR (Antibody, IgM):ti,ab,kw OR (IgM):ti,ab,kw OR (IgM Antibody):ti,ab,kw OR (IgM2):ti,ab,kw OR (IgM1):ti,ab,kw with Cochrane Library publication date Between Jan 1989 and May 2023 2309
#15 #1 OR #2 669
#16 #3 OR #4 2267
#17 #5 OR #6 3018
#18 #7 OR #8 556394
#19 #9 OR #10 16753
#20 #11 OR #12 11444
#21 #13 OR #14 2566
#22 MeSH descriptor: [Sensitivity and Specificity] explode all trees 19949
#23 (efficacy*):ti,ab,kw with Cochrane Library publication date Between Jan 1989 and May 2023 420530
#24 MeSH descriptor: [Data Accuracy] explode all trees 120
#25 #15 OR #16 with Cochrane Library publication date Between Jan 1989 and May 2023 2586
#26 #17 OR #18 OR #19 OR #20 OR #21 with Cochrane Library publication date Between Jan 1989 and May 2023 573246
#27 #22 OR #23 OR #24 with Cochrane Library publication date Between Jan 1989 and May 2023 438778
#28 #25 AND #26 AND #27 with Cochrane Library publication date Between Jan 1989 and May 2023 482

Search Database: Web of science

Publication date: January 1, 1989, to May 31, 2023.

Search Strategy:1: TI=(Hepatitis Delta Viruses) OR TI=(Hepatitis D Virus) OR TI=(Hepatitis D Viruses) OR TI=(Delta Virus) OR TI=(Delta Viruses) OR

AB=(Hepatitis Delta Viruses) OR AB=(Hepatitis D Virus) OR AB=(Hepatitis D Viruses) OR AB=(Delta Virus) OR AB=(Delta Viruses) OR TS=(Delta Hepatitis) OR TS=(Delta Infection) OR AB=(Delta Hepatitis) OR AB=(Delta Infection) and Preprint Citation Index (Exclude – Database) Timespan: 1989-01-01 to 2023-05-31 Results: 57240

2: TI=(Serologic Tests) OR TI=(Serological Tests) OR TI=(Serological Test) OR TI=(Serologic Test) OR AB=(Serologic Tests) OR AB=(Serological Tests) OR AB=(Serological Test) OR AB=(Serologic Test) and Preprint Citation Index (Exclude – Database) Timespan: 1989-01-01 to 2023-05-31 Results: 46842

3: TI=(Diagnoses) OR TI=(Diagnose) OR TI=(Diagnoses and Examinations) OR TI=(Examinations and Diagnoses) OR TI=(Diagnoses and Examination) OR TI=(Examination and Diagnoses) OR AB=(Diagnoses) OR AB=(Diagnose) OR AB=(Diagnoses and Examinations) OR AB=(Examinations and Diagnoses) OR AB=(Diagnoses and Examination) OR AB=(Examination and Diagnoses) and Preprint Citation Index (Exclude – Database) Timespan: 1989-01-01 to 2023-05-31 Results: 3171076

4: TI=(Enzyme Linked Immunosorbent Assay) OR TI=(ELISA) OR TI=(Enzyme-Linked Immunosorbent Assays) OR AB=(Enzyme Linked Immunosorbent Assay) OR AB=(ELISA) OR AB=(Enzyme-Linked Immunosorbent Assays) and Preprint Citation Index (Exclude – Database) Timespan: 1989-01-01 to 2023-05-31 Results: 311968

5: TI=(IgG Antibody) OR TI=(IgG) OR AB=(IgG Antibody) OR AB=(IgG) and Preprint Citation Index (Exclude – Database) Timespan: 1989-01-01 to 2023-05-31 Results: 160769

6: TI=(IgM) OR TI=(IgM Antibody) OR AB=(IgM) OR AB=(IgM Antibody) and Preprint Citation Index (Exclude – Database) Timespan: 1989-01-01 to 2023-05-31 Results: 70942

7: TI=(Sensitivity and Specificity) OR AB=(Sensitivity and Specificity) and Preprint Citation Index (Exclude – Database) Timespan: 1989-01-01 to 2023-05-31 Results: 340650

8: TI=(efficacy) OR AB=(efficacy) and Preprint Citation Index (Exclude – Database) Timespan: 1989-01-01 to 2023-05-31 Results: 1456383

9: TI=(Accuracy) OR AB=(Accuracy) and Preprint Citation Index (Exclude – Database) Timespan: 1989-01-01 to 2023-05-31 Results: 2657641

10: #1 AND (#2 OR #3 OR #4 OR #5 OR #6) AND (#7 OR #8 OR #9) and Preprint Citation Index (Exclude – Database) Timespan: 1989-01-01 to 2023-05-31 Results: 792
